# Sperm Heterogeneity Accounts for Sperm DNA Methylation Variations Observed in the Caput Epididymis, Independently From DNMT/TET Activities

**DOI:** 10.3389/fcell.2022.834519

**Published:** 2022-03-22

**Authors:** Hong Chen, Marie-Pier Scott-Boyer, Arnaud Droit, Claude Robert, Clémence Belleannée

**Affiliations:** ^1^ Faculty of Medicine, Université Laval, Quebec, QC, Canada; ^2^ Center for Research in Reproduction, Development and Intergenerational Health, Quebec, QC, Canada; ^3^ Faculty of Animal Sciences, Université Laval, Quebec, QC, Canada

**Keywords:** DNA methylation, methyltransferase, epididymis, spermatozoa, RRBS (reduced representation bisulphite sequencing), epigenetics

## Abstract

Following their production in the testis, spermatozoa enter the epididymis where they gain their motility and fertilizing abilities. This post-testicular maturation coincides with sperm epigenetic profile changes that influence progeny outcome. While recent studies highlighted the dynamics of small non-coding RNAs in maturing spermatozoa, little is known regarding sperm methylation changes and their impact at the post-fertilization level. Fluorescence-activated cell sorting (FACS) was used to purify spermatozoa from the testis and different epididymal segments (i.e., *caput, corpus and cauda*) of CAG/su9-DsRed2; Acr3-EGFP transgenic mice in order to map out sperm methylome dynamics. Reduced representation bisulfite sequencing (RRBS-Seq) performed on DNA from these respective sperm populations indicated that high methylation changes were observed between spermatozoa from the *caput* vs. testis with 5,546 entries meeting our threshold values (q value <0.01, methylation difference above 25%). Most of these changes were transitory during epididymal sperm maturation according to the low number of entries identified between spermatozoa from *cauda* vs. testis. According to enzymatic and sperm/epididymal fluid co-incubation assays, (de)methylases were not found responsible for these sperm methylation changes. Instead, we identified that a subpopulation of *caput* spermatozoa displayed distinct methylation marks that were susceptible to sperm DNAse treatment and accounted for the DNA methylation profile changes observed in the proximal epididymis. Our results support the paradigm that a fraction of *caput* spermatozoa has a higher propensity to bind extracellular DNA, a phenomenon responsible for the sperm methylome variations observed at the post-testicular level. Further investigating the degree of conservation of this sperm heterogeneity in human will eventually provide new considerations regarding sperm selection procedures used in fertility clinics.

## Introduction

During sperm differentiation and post-testicular maturation in the epididymis, the paternal genome is subjected to epigenetic changes that influence proper embryo development and may have consequences on progeny health (Review Chen et al., 2021) (Champroux et al., 2018; Sharma, 2019; Chen et al., 2021). While sperm epigenetic changes, particularly DNA methylation programming, have been exhaustively investigated during spermatogenesis, the epigenetic dynamics and mechanisms occuring at the post-testicular level remain poorly explored (Ariel et al., 1994; Ben Maamar et al., 2019; Galan et al., 2021).

DNA methylation is a widespread epigenetic mark targeting CpG dinucleotides (5-methylcytosine, 5mC) along the mammalian genome. While these chemical tags control chromatin structure and gene expression during development, they are dynamic and undergo two large-scale demethylation waves: one in the male germline and the second after fertilization. As a consequence, most methylation marks inherited from the mature male gamete will be erased in the developing embryo, with the exception of differentially methylated regions (DMRs) of imprinted genes, repetitive elements such as transposons, and some escape genes (Law and Jacobsen, 2010; Smith et al., 2012; Smith and Meissner, 2013; Tang et al., 2015). Thus, while most DNA methylation changes occuring in the sperm genome before fertilization may have no or little consequences after fertilization, some are retained and may be instrumental to proper embryo development.

Due to the importance of methylation marks in the control of developmental gene expression, addition and removal of a methyl group to the 5-position of cytosine (5mC) is tightly controlled by DNA methyltransferases (DNMTs) and ten-eleven translocation (TET) methylcytosine dioxygenases, respectively (Li and Zhang, 2014; Rasmussen and Helin, 2016; Edwards et al., 2017). In mammals, the DNA methylation machinery comprises DNMT1, DNMT3a, DNMT3b, DNMT3c and DNMT3L (Kaneda et al., 2004; Li and Zhang, 2014; Barau et al., 2016). Conversely, replacement of 5mC with unmodified cytosine occurs under the control of TET1, TET2, and TET3, which share similar structures and catalytic activities (Rasmussen and Helin, 2016). While the presence of DNMT1/3a/3b and TET1/2/3 has been described in mature/ejaculated spermatozoa (Marques et al., 2011; Ni et al., 2016), their activity level and potential contribution to *de novo* sperm DNA methylation profiling remains to be determined.

After spermatogenesis and prior to entering the epididymis where motility and fertilizing abilities are acquired, spermatozoa carry an ultra condensed genome due to the replacement of most histones by protamine. Until recently, the study of the epididymis has focussed on its contribution to sperm quality control (i.e., its ability to protect against pathogens and to potentially eliminate defective spermatozoa), and sperm maturation through the regulation of physiological, biochemical and functional sperm features (Sutovsky, 2003; Axner, 2006; Cornwall et al., 2007; Ramos-Ibeas et al., 2013; Domeniconi et al., 2016; Sullivan and Mieusset, 2016; Zhou et al., 2018; James et al., 2020). Adding to these important contributions are recent studies that shed light on the role played by the epididymis in the composition of the sperm epigenome in response to diverse environmental cues (e.g., stress, alcohol consumption, diet, chemicals) (Sharma, 2019; James et al., 2020; Chen et al., 2021). While these epigenetic sperm modifications mostly refer to the transfer of small non-coding RNA (microRNA and tRNA-derived fragments) via epididymis-derived extracellular vesicles, a limited number of studies have investigated the dynamics of sperm DNA methylation occuring during post-testicular sperm maturation (Ariel et al., 1994; Ben Maamar et al., 2019; Galan et al., 2021). Years before the advancement of genome-wide DNA methylation profiling methods, Ariel et al. showed that Pgk-2, ApoA1, and Oct-3/4 loci were subjected to remethylation in the different segments of the epididymis, suggesting that a wave of sperm DNA reprogramming might occur at the post-testicular level through DNA methylation (Ariel et al., 1994; McCarrey et al., 2005). Moreover, Ben Maamar et al. showed that exposure to DDT and vinclozolin triggered sperm DNA methylation changes in the *caput* and *cauda* epididymidis, suggesting that the post-testicular sperm methylome is regulated by environmental conditions (Ben Maamar et al., 2019). While these seminal studies raised questions regarding the mechanisms by which enzymes can access the highly compacted sperm genome to catalyze its (de)methylation, a recent genome-wide study from Galan *et al.* suggested that the transient DNA methylation changes observed in the proximal region of the epididymis was due to the release of cell-free DNA by somatic cells (Galan et al., 2021).

In the present study we took advantage of the CAG/su9-DsRed2, Acr3-EGFP double transgenic mouse model developed by Hasuwa et al. to isolate a pure population of fluorescent spermatozoa devoid of somatic cell (Hasuwa et al., 2010). Genome-wide reduced representation bisulfite sequencing (RRBS) performed on fluorescence-activated sorted spermatozoa (FACS) from the testis and different segments of the epididymis, i.e., *caput*, *corpus* and *cauda*, supported the existence of a variable methylation profiling at the post-testicular level, with a major and transient change observed in the proximal segment of the epididymis. Subsequent *in vitro* assays and pyrosequencing on spermatozoa were performed to assess the possible contributions of DNMT/TET enzymes and sperm heterogeneity to these changes.

## Methods

### Mice

Testes and epididymis were collected from 10 to 12-week-old CAG/su9-DsRed2, Acr3-EGFP transgenic mice (MTA to EB), and C57BL/6 wildtype mice. Colonies were reproduced and maintained in the elite animal facility of CHU de Quebec Research Center. This study was approved by the ethical committee of the Institutional Review Board of the Centre Hospitalier Universitaire de Québec (CHUQ; CPAC licenses 16-050-4 and 16-051-4, C. Belleannée) and was conducted in accordance with the requirements defined by the Guide for the Care and Use of Laboratory Animals.

### Harvest of Spermatids and Epididymal Spermatozoa

**FIGURE 1:**
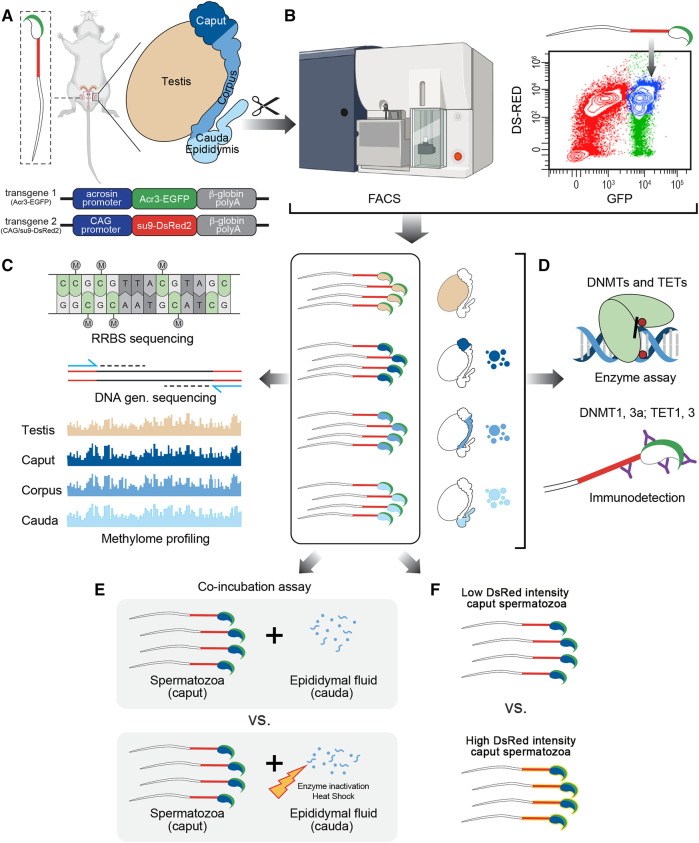
Experimental design followed in this study. **(A)** Male reproductive system was collected from CAG/su9-DsRed2, Acr3-EGFP transgenic mice and dissected into regions corresponding to the testis, *caput*, *corpus*, and *cauda* epididymidis. **(B)** Spermatozoa were isolated from each region by fluorescence-activated cell sorting (FACS) based on their endogenous fluorescence. **(C)** Following DNA extraction, methylation profiling was performed by reduced representation bisulfite sequencing (RRBS)—Illumina next-generation sequencing and validated by pyrosequencing. **(D)** The detection and activity levels of methyltransferases (DNMTs) and demethylases (TETs) were performed by immunodetection (western blot and immunofluorescence staining) and enzyme assays from spermatozoa and epididymal fluid samples. **(E)** The effect of epididymal fluid on sperm methylome dynamics was assessed by *in vitro* co-incubation assays. **(F)** DNA methylation profile was analyzed on sperm population with low *vs.* high DsRed intensity level.

CAG/su9-DsRed2, Acr3-EGFP transgenic mice were anesthetized by ketamine-xylazine and subjected to transcardiac perfusion with phosphate-buffered saline (PBS; 137 mM NaCl, 3 mM KCl, 8 mM Na_2_HPO_4_, and 1.5 mM KH_2_PO_4_) to prevent blood contamination in the samples. After complete PBS replacement, the testis and three principal epididymal segments (i.e., *caput*, *corpus*, and *cauda*) were dissected for sperm collection and analysis, as described in the experimental design (Figure 1). A syringe was inserted into the proximal portion of the vas deferens for collection of spermatozoa and fluid from the cauda epididymidis by retrograde intraluminal perfusion with PBS at a rate of 10 μl/min under the control of a syringe pump. Spermatozoa were then isolated from the epididymal fluid by centrifugation at 2000 g for 10 min at 4°C. Epididymal fluid was stored at −80°C for later use. To isolate spermatozoa from the upstream segments, testicular and epididymal tissues (*caput* and *corpus*, separately) were cut into 2 mm^3^ pieces. Tissues were placed in a Medimachine System (BD Biosciences, San Jose, Canada) with 1 ml PBS containing 0.2% 2-naphthol-6,8-disulfonic acid to obtain a single-cell suspension. The cellular suspension was filtered twice through a 50 μm CellTrics filter (Sysmex, United States) and spermatozoa were isolated from somatic cells by flow cytometry at the Flow Cytometry Core Facility of the CHU de Quebec Research Center.

### Fluorescence-Activated Cell Sorting of Spermatozoa

Cells were sorted with a 70 µm nozzle on a BD Aria Fusion cell sorter (BD Biosciences, San Jose, Canada). Spermatozoa were purified based on their endogenous fluorescence, i.e., double-positive for GFP and DsRed (GFP^+^ DsRed^+^). A first gate was settled on GFP-positive events to facilitate the selection of our cells of interest. From those cells, doublets were gated out and DsRed-positive spermatozoa were isolated and sorted into 15 ml Falcon tubes containing PBS. *Cauda* spermatozoa from C57BL/6 wild-type mice and CAG/su9-DsRed2, Acr3-EGFP transgenic mice were used as negative and positive controls, respectively. The purity of spermatozoa isolated by FACS was assessed under fluorescence microscope by counting more than 100 cells per sample. Alternatively, *Caput* spermatozoa were further discriminated by FACS according to their endogenous DsRed intensity (High DsRed vs. Low Ds Red). Prior to sperm DNA extraction and pyrosequencing, half of each subpopulation (0.5 million of spermatozoa) were treated with DNAse I (Qiagen, United States) following the manufacturer’s instructions.

### Spermatic Genome Isolation

Spermatozoa were incubated overnight at 55°C in DNA lysis buffer (100 mM Tris HCl (pH 8.5), 5 mM EDTA, 0.2% SDS, 200 mM NaCl, 100 mM DTT, 300 μg/ml proteinase K) to ensure the dissociation of protamines. Spermatic genome extraction was performed following the standard phenol:chloroform protocol. In brief, a 1:1 volume of phenol:chloroform: isoamyl alcohol (25:24:1, Invitrogen) was added to each sperm suspension. The aqueous fraction was collected and precipitated with 250 mM NaCl, a 2.5:1 ratio of ethanol, and 1 µl 20 μg/μl glycogen (Invitrogen, United States) after vortexing and a 5-min centrifugation at maximum speed. Spermatic DNA was precipitated overnight at −20°C, and washed twice with 70% ethanol. Finally, DNA was resuspended in 30 µl DNAse-free water. The quantity and quality of our samples were assessed by Qubit kit (ThermoFisher, Canada) and the Agilent 2100 Bioanalyzer Systems (Agilent, United States).

### Reduced Representation Bisulfite Sequencing

Diagenode Premium RRBS kit (Diagenode, Belgium) was used following the manufacturer’s instructions. Digested DNA was purified using AMPure XP Beads and bisulfite was converted with the BS conversion reagent supplied in the kit. The library was generated by large-scale PCR amplification and purified with AMPure XP Beads. Library quality control was performed on a bioanalyzer system (Agilent, United States) and compared with the expected results for mouse libraries obtained following the manufacturer’s instructions. Libraries were sequenced on an Illumina 2500 Rapid Run apparatus at the Genomic Core Facility of the CHU de Quebec Research Center. Quality control was performed with fastQC (https://www.bioinformatics.babraham.ac.uk/projects/fastqc/). Low-quality reads and adapters were removed with *Trim Galore* (version 0.4.1) (Bolger et al., 2014). The remaining reads were aligned to the *Mus musculus* reference genome (mouse genome GRcm38) with Bismark (version 0.16.1) (Krueger and Andrews, 2011). Paired methylation profile comparisons were performed between *caput* spermatozoa, *corpus* spermatozoa, and *cauda* spermatozoa *vs.* testicular spermatozoa, respectively. Differential methylation sites (DMS) and differential methylation regions (DMR) were identified with the R package methylKit, which used logistic regression to calculate *p*-values (Akalin et al., 2012). The *p*-values were adjusted to q-values using Sliding Linear Model (SLIM) method. DMS and DMR were identified by using methylKit with a q-values threshold of 0.01 and a methylation difference above 25%. In addition, the RepeatMasker annotation downloaded from the UCSC website was used to analyze repetitive DNA (http://www.repeatmasker.org). The raw data used in this study are publicly available through the Gene Expression Omnibus repository website (https://www.ncbi.nlm.nih.gov/geo/) GSE191051.

### Functional Enrichment Analysis

Genes presenting with differentially methylated regions in their promoter regions were analyzed using the Database for Annotation, Visualization and Integrated Discovery database (DAVID bioinformatics resources 6.8, NIAID/NIH) to determine their biological functions and pathways. The GO and pathway enrichment were performed in GO enrichment and Kyoto Encyclopedia of Genes and Genomes (KEGG) enrichment analysis program. The promoter was defined ±2 kb from the transcription start site (TSS) of the gene using the mouse genome GRcm38 as a reference and a *p* value <0.05.

### Pyrosequencing

Primers were designed for genes of interest using PyroMark Assay Design software (Qiagen, United States) (Supplementary Table S1 **)**. Bisulfite conversion of 40 ng of spermatic DNA was prepared by EZ DNA Methylation-Gold kit (Zymo Research, United States). Twenty ng of bisulfite converted DNA were amplified for pyrosequencing using Pyromark Q24 kit (Qiagen, United States) and Pyromark Q24 Vacuum workstation (Qiagen, United States) according to the manufacturer’s instructions.

### Immunohistochemistry

Testis and epididymis were collected from C57BL/6 mice, fixed in 4% PFA and embedded in paraffin. Five-μm paraffin sections were deparaffinized in xylene and antigen was unmasked using citrate buffer (10 mM, pH 6.0) for 10 min at 110°C. Protein binding sites were blocked with 0.5% bovine serum albumin (BSA) (Sigma, Canada) for 1 h. Primary antibodies diluted in DAKO solution (Agilent, United States) were incubated overnight at 4°C (Supplementary Table S2). After washing twice with PBS, the secondary biotinylated antibodies were incubated for 1 h at room temperature **(** Supplementary Table S2 **)**. ABC elite reagent (Vector Laboratories, Inc. Burlingame, Canada) was applied to the slides following staining with 3-amino-9-ethylcarbazole (AEC) (Sigma, United States) and hematoxylin (Fisher Chemical, Canada). After addition of aqueous mounting medium (Sigma, Canada), tissue sections were observed under a Zeiss Axioskop 2 Plus microscope linked to a digital camera from Qimaging. Images were captured using the QCapture Pro (Qimaging Instruments).

### Immunofluorescence

Spermatozoa from the *caput, corpus*, and *cauda* were spread and air dried on fibronectin-coated slides for 30 min. After treatment with 2 mM DTT and 0.5% Triton X-100 for 45 min, cells were fixed with 4% PFA. After treatment with citrate buffer (10 mM, pH 6.0) for 10 min at 110°C, protein binding sites were blocked with 1% BSA for 30 min. Primary antibodies diluted in DAKO solution were incubated overnight at 4°C **(** Supplementary Table S2 **)**, followed by the incubation with the secondary antibody diluted in PBS for 1 h at room temperature **(** Supplementary Table S2 **)**. After mounting in an aqueous medium (Sigma), images were obtained using an Olympus IX80 confocal microscope.

### Protein Extraction

Testis, epididymis tissues and spermatozoa were disrupted in RIPA lysis buffer (150 mM NaCl, 50 mM Tris, 0.1% SDS, 1% Triton, 0.5% deoxycholate, 1 mM EDTA, pH 7.4) in the presence of a proteinase inhibitor cocktail (cOmplete, Mini, EDTA-free Protease Inhibitor Cocktail, Roche, CANADA) using FastPrep-24 system (MP Biomedical, Inc., DE). Tissues underwent two 30-s rounds of homogenization. The debris were discarded after centrifugation at 12,000 g for 15 min at 4°C.

### Nuclear Protein Extraction

The nuclear protein fraction was extracted from spermatozoa using the Nuclear Extraction Kit following the manufacturer’s recommendations (Abcam, United States). In brief, 2 × 10^6^ testicular, *caput*, *corpus*, and *cauda* spermatozoa were suspended in 100 µl of 1× Pre-Extraction Buffer supplied with the kit. After a 10-min incubation on ice, the cell suspension was vortexed vigorously for 10 s and centrifuged at 10,000 g for 1 min. The cytoplasmic fraction was carefully removed from the nuclear pellet and stored at −20°C for later use. Twenty μl of Extraction Buffer containing DTT and Protease Inhibitor Cocktail were added to the nuclear pellet and incubated on ice for 15 min with a 5-s vortex step every 3 min. The suspension was collected after centrifugation at 14,000 g for 15 min at 4°C. Protein concentration was determined using the Bradford reagent (Biorad, CANADA) following the standard protocol.

### Isolation of Extracellular Vesicles From the Epididymal Fluid

Epididymal fluid was isolated from the *cauda* epididymis by retrograde intraluminal perfusion and centrifugation, as described above. Epididymal fluid from the *caput* and *corpus* epididymidis was collected following excision of the tissues and incubation in 100 μl PBS under agitation, as previously described (Nixon et al., 2019). Fluid suspension was centrifuged at 12,000 g for 20 min to eliminate cellular debris. Finally, epididymal fluids collected from the different segments were subjected to ultracentrifugation at 100,000 g for 2 h at 4°C to isolate extracellular vesicles.

### Western Blot

Proteins extracts were boiled in Laemmli sample buffer containing 2% β-mercaptoethanol at 95°C for 5 min. Denatured proteins were loaded onto a 5–15% gradient SDS-polyacrylamide gel, followed by transfer onto nitrocellulose membranes (0.45 µm, Bio-Rad) using the Trans-Blot Turbo system (Bio-Rad). Membranes were blocked in 5% milk diluted in PBS with Tween 20 (PBST, PBS with 0.05% Tween 20) at room temperature for 1 h and incubated overnight at 4°C with primary antibody diluted in 5% BSA **(** Supplementary Table S2). After three washes in PBS, membranes were incubated with the appropriate secondary antibody conjugated to horseradish peroxidase at room temperature for 1 h **(** Supplementary Table S2 **)**. Detection was performed with the Clarity or Clarity Max Western ECL substrate (BioRad, Canada) on a ChemiDoc MP Imaging System (BioRad, Canada). Quantification was performed on target signal intensity normalized to controls, β-actin and HDAC I, using the ImageLab system (BioRad, Canada).

### Enzymatic Activity Assay

DNMT and TET activities were assayed on 5–10 ng of testicular, *caput*, *corpus* and *cauda* spermatic nuclear extractions, in addition to epididymal fluids from *caput*, *corpus*, and *cauda*. The negative control was prepared by boiling a fraction of epididymal fluid at 95°C for 10 min to inactivate enzymatic activity. Purified DNMT and TET enzymes were used as positive controls. The assay was performed using the DNMT and TET colorimetric activity assays (Abcam, Canada) following the manufacturer’s instructions. Signal was quantified on a Spark™ 10M system (TECAN, Switzerland) at 450 nm with a reference wavelength at 655 nm.

### Epididymal Fluid–Spermatozoa Co-Incubation Assays

For each co-incubation assay, *caput* spermatozoa and epididymal fluid from the *cauda* epididymidis were pooled from 4 mice. C*aput* spermatozoa (5 × 10^5^) were incubated for 2 h at 37°C in 100 μl *cauda* epididymal fluid at a protein concentration of 1 ng/μl. The negative control comprised 5 × 10^5^ caput spermatozoa incubated with 100 μl enzyme-inactivated epididymal fluid (described above). After co-incubation, cells were collected by centrifugation at 2000 g for 10 min at room temperature prior to DNA extraction. Methylation of genes of interest was assessed by pyrosequencing, as described above. This experiment was repeated four times.

### Statistical Analyses

Statistical *t*-test analyses were performed by using Graphpad Prism 7 software. All results are presented as mean ± SEM or percentages (%) from at least three independent experiments.

## Results

### Sorting of Spermatozoa From CAG/su9-DsRed2, Acr3-EGFP Transgenic Mice Prevents Contamination From Somatic Cells

**FIGURE 2:**
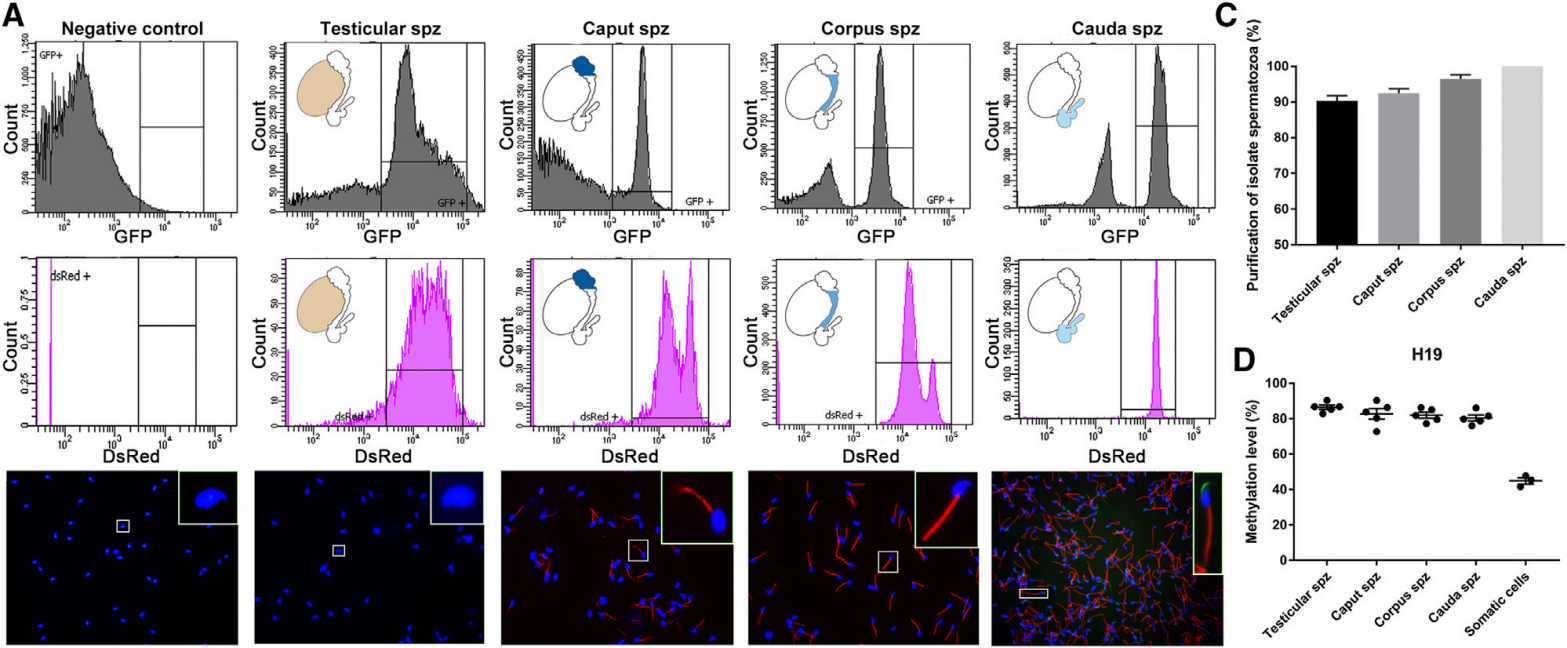
Isolation of testicular, *caput*, *corpus* and *cauda* spermatozoa by fluorescence-activated cell sorting (FACS). **(A)** Spermatozoa were isolated by FACS based on their endogenous EGFP and dsRed fluorescence. Spermatozoa from wild-type mice and spermatozoa collected by intraluminal perfusion from the cauda epididymidis were used as a negative control and positive control, respectively. Purification levels of spermatozoa were assessed by cell counting under a fluorescence microscope **(B,C)** and by H19-IGF2 methylation pyrosequencing **(D)**. Somatic cells isolated from the tail of transgenic mice were used as a reference. Spz: spermatozoa sample.

Spermatozoa were isolated from the different anatomical regions (i.e., testis, caput, corpus, and cauda) of 10–12-week-old CAG/su9-DsRed2, Acr3-EGFP transgenic mice by FACS. Sorting parameters were determined from the dual endogenous fluorescence of CAG/su9-DsRed2 and Acr3-EGFP cauda spermatozoa, which were isolated by intraluminal perfusion to minimize somatic cell contaminations. Only the events presenting the strongest GFP and DsRed signals where selected (Figure 2A), representing 9.2, 5.9 and 22.9% of total events in the testis, *caput* and *corpus* epididymis, respectively (not shown). According to DsRed signal intensity, two populations of spermatozoa could be distinguished in the *caput* epididymidis and, to a lower extent, in the *corpus* epididymidis (Figure 2A). As observed by fluorescence microscopy (Figure 2B), the purification yield was 90 ± 5%, 95 ± 5%, and 98 ± 2% in these segments, and no somatic cell contamination was observed in the *cauda* epididymidis (Figure 2C **)**. Approximately 1 million spermatozoa were retrieved from the distinct anatomical segments isolated from a pool of four mice. Since the H19 promoter region is hypermethylated in male germinal cells (Bartolomei et al., 1991; Zhang and Tycko, 1992), this specific site was pyrosequenced to assess the purity level of sorted germinal cells (Figure 2D). While methylation of the H19 promoter was 42 ± 3% in control somatic cell DNA extracts, it was twice as high 87 ± 1%, 82 ± 4%, 82 ± 2%, and 80 ± 2% in DNA extracts from sorted testicular, *caput*, *corpus*, and *cauda* spermatozoa, respectively. This supports the purity of the sperm DNA extracts used for subsequent reduced representation bisulfite sequencing (RRBS) methylation profiling.

### Distinct DNA Methylation Profiles are Found in the Discrete Segments of the Epididymis

**FIGURE 3:**
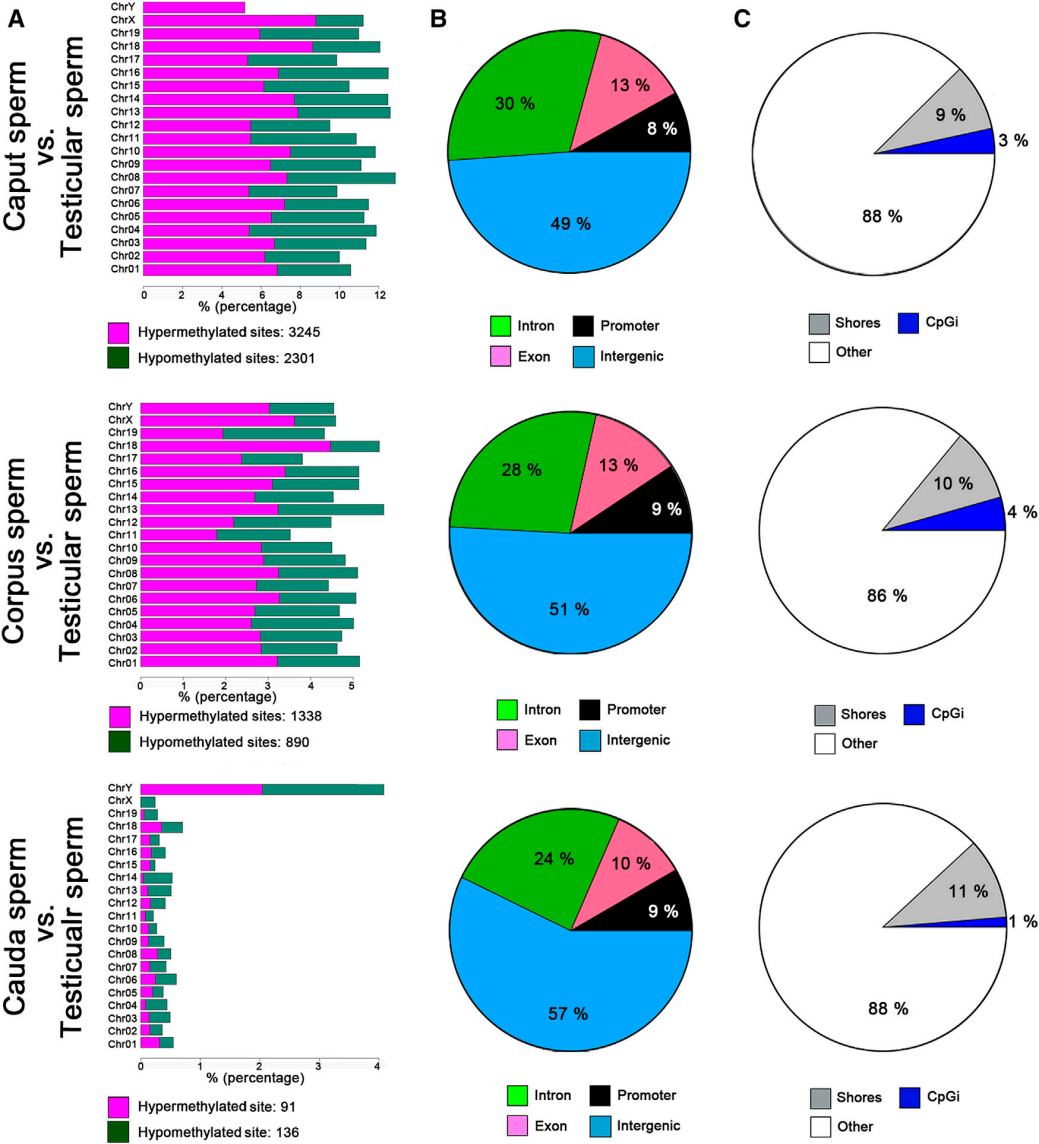
Global changes in sperm DNA methylation profiles at the post testicular level. **(A)** Distribution of CpG hypermethylated (purple) and hypomethylated sites (green) within each chromosome. **(B)** Pie charts illustrating the proportions of CpGs covered by RRBS in regions annotated as introns (green), promoter regions (black), exons (purple), or intergenic regions (blue). **(C)** Pie chart illustrating the proportions of CpGs covered by RRBS in regions annotated as CpG islands (blue), CpG shores (gray) and other regions (white). Results are representative of three independent experiments.

Genome-wide RRBS was used to investigate the methylation profile of spermatozoa from different post-testicular regions. This analysis featured approximately 1% of the sequenced mouse genome, covering approximately 35% of cytosine methylation rate in the CpG context. An inter-sample correlation coefficient of 0.96 was observed among sample triplicates. Comparison of the methylation profile of spermatozoa from different epididymal segments (i.e., *caput*, *corpus*, and *cauda* spermatozoa) with testicular spermatids/spermatozoa, indicated that the sperm cytosine methylation profile was variable at the post-testicular level (Figure 3A), with an equal proportion of hypermethylated and hypomethylated sites in autosomal chromosomes. The highest changes were identified between the *caput* epididymis *vs.* testis with the detection of 5,546 differentially methylated CpG sites (DMS) (q value <0.01, methylation difference above 25%). The number of differentially methylated CpG sites (DMS) reduced gradually to 2228 in the *corpus* vs. testis, and to 228 in the *cauda* vs. testis. Among imprinting genes whose epimutations are associated with embryonic disorders, H19, IGF2, MEST, KCNQ1, SNRPN displayed consistent methylation levels in spermatozoa from the different segments of the epididymis (not shown). Furthermore, half of the methylation changes between testicular and *caput* spermatozoa where observed in the intergenic regions and around 25, 13, and 9% in the intron, exon, and promoter regions, respectively (Figure 3B). This proportion was maintained in the other epididymal segments. While these methylation variations were evenly distributed in chromosome 1 to 19, and X chromosome, we identified four main genes on the Y chromosome including RNA-binding motif on Y chromosome (RMBY), Gm20854, G530011O06Rik and spermiogenesis-specific transcript on the Y 2 (ssty2) that present a significant change during the post-testicular maturation (Supplementary Figure S1). Furthermore, 653 DMS, 263 DMS, and 51 DMS from *caput*, *corpus*, and *cauda* spermatozoa were found in repetitive regions, representing 11.7, 11.8, and 22.6% of total DMS, respectively. This includes DMS from long interspersed nuclear elements (LINEs) [*Caput* vs. testis: 149 (22.5%), *Corpus* vs. testis: 75 (28.7%), *Cauda* vs. testis: 19 (37.3%)], long terminal repeats (LTRs) [*Caput* vs. testis: 263 (39.7%), *Corpus* vs. testis: 105 (40.2%), *Cauda* vs. testis: 23 (45.1%)] short interspersed nuclear elements (SINEs) [*Caput* vs. testis: 233 (35.2%), *Corpus* vs. testis: 75 (28.7%), *Cauda* vs. testis: 9 (17.6%)], and in satellites [*Caput* vs. testis: 17 (2.6%), *Corpus* vs. testis: 8 (2.3%), *Cauda* vs. testis: 0 (0%)] (Supplementary Figure S2).

### Validation of RRBS Data by Pyrosequencing

**FIGURE 4:**
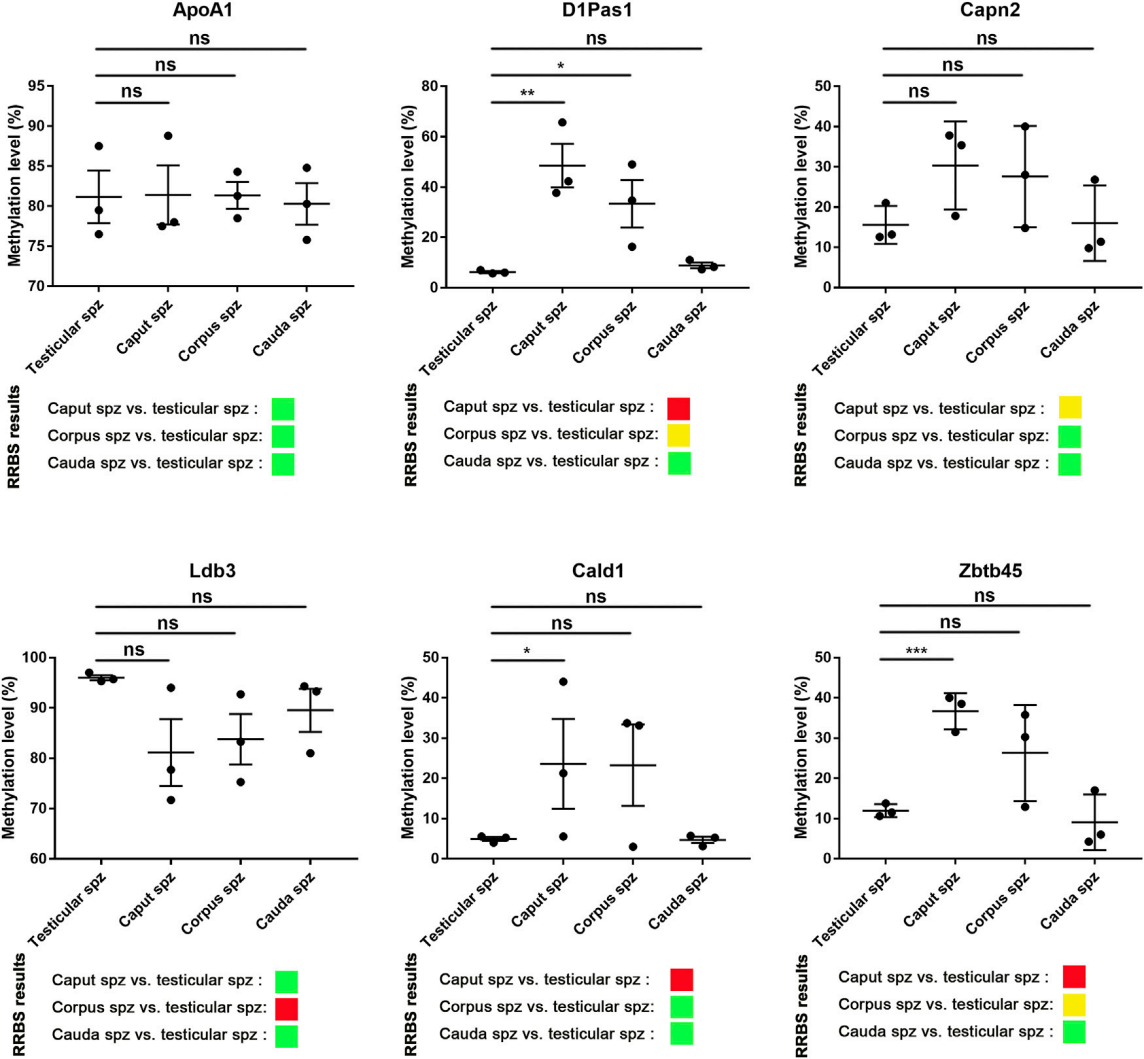
Pyrosequencing validation of RRBS data. Six spermatic DNA loci that exhibited significant methylation changes during post-testicular maturation were validated by pyrosequencing. For each locus, 3 samples of testicular, *caput*, *corpus*, and *cauda* spermatozoa were used. For comparison, differential methylation profiles obtained by RRBS are shown. Green, yellow, and red colors represent methylation changes of <25%, 25–50%, and >50%, respectively. *, **, and ** represent significant differences *p* < 0.05, <0.005, and <0.001 respectively.

According to RRBS methylation profiling, 79% of methylation changes were identified in spermatozoa from the *caput* epididymis **(** Supplementary Figure S3), with the top 10 listed in Table 1. Among these genes, D1Pas1, Capn2, Ldb3, Cald1, and Zbtb45 were selected for validation of methylation changes by pyrosequencing **(** Figure 4). ApoA1 was included in the analysis as a gene previously shown to be methylated during epididymal sperm maturation (Ariel et al., 1994). Pyrosequencing analysis validated the changes in sperm methylation during the post-testicular maturation observed by RRBS, with major methylation changes detected in *caput* spermatozoa compared with other segments (Figure 4 **)**. No significant changes were observed between testicular and *cauda* spermatozoa for any of the selected genes. Whereas D1Pas1, Cald1, and Zbtb45 methylation sites all followed a transitory hypermethylation in the *caput* segment of the epidiymidis, only Ldb3 presented transitory hypomethylation. Furthermore, no significant methylation changes were detected by pyrosequencing or RRBS for ApoA1 at the post-testicular level (Figure 4).

**TABLE 1:** The top 10 most differentially methylated regions (DMRs) identified from caput vs. testicular spermatozoa.

Gene	Location	Methylation difference	q-value	Number of DMS	Function
Cald1	Chr6: 34755701-34755800	78.8%	5.5e-114	7	Actin, calmodulin, and myosin binding
Zbtb45	Chr7: 13007801-13007900	50.8%	1.2e-45	7	Transcriptional regulation
Susd1	Chr4: 59360901-59361000	−36.7%	1.7e-82	6	Calcium ion binding
Ano8	chr8: 71481601-71481700	52.0%	4.1e-45	5	Intracellular calcium activated chloride channel activity
Nfatc2	Chr2: 168571001-168571100	50.1%	4.7e-30	5	Inducing of gene transcription during the immune response
Irf8	chr8: 120739801-120739900	−45.7%	1.7e-26	5	DNA-binding transcription factor activity
Ankrd36	Chr6: 5634501-5634600	35.4%	3.4e-17	5	Protein binding
Notch2	Chr3: 98024501-98024600	−33.6%	1.3e-20	5	Receptor for membrane bound ligands
1700123L14Rik	Chr6: 96164801-96164900	76.6%	5.5e-109	4	mRNA transport
Ccbe1	chr18: 66084701-66084800	66.4%	4.2e-64	4	Calcium ion, collagen, and protease binding

### Differentially Methylated Sites From Promoter Regions are Associated With Developmental Processes

**FIGURE 5:**
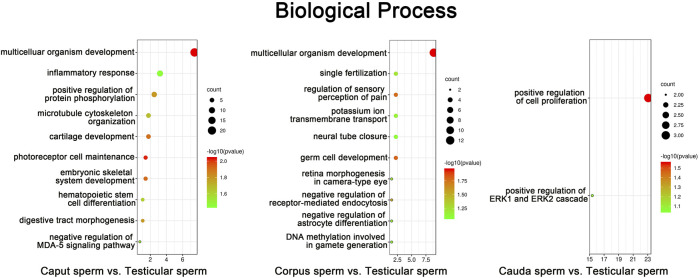
Gene Ontology (GO) enrichment analysis of sperm methylation changes identified during post-testicular maturation. CpG methylation sites from promoter regions that presented the highest significant changes among spermatozoa from different segments (% change >25, Q value <0.05) were submitted to GO analysis. The top ten most significant enriched biological process GO terms are presented (*p* < 0.05). The color and size of each point represents the -log10 (FDR) values and enrichment scores, respectively. A higher -log_10_ (FDR) value and enrichment score represents a greater degree of enrichment.

Annotated genes associated with differentially methylated sites in promoter regions according to RRBS data were subjected to gene ontology (GO) analysis using the DAVID program. *In silico* analysis revealed the top ten GO terms associated with genes methylated during sperm post-testicular maturation (*p* < 0.05) (Figure 5). From both *caput* and *corpus* epididymal segments, the top-ranked biological function was “multicellular organism development”. For instance, GO terms including “microtubule cytoskeleton organization”, “cartilage development”, and “embryonic skeletal development” were among the most significant GO terms found associated with *caput* methylation changes. Among functions associated with methylation changes from the *corpus* spermatozoa were “fertilization”, “germ cell development” and “DNA methylation involved in gamete generation”. While a limited number of changes were detected in spermatozoa from the *cauda* epididymidis, these methylation marks were mainly predicted to be associated with “regulation of cell proliferation”.

### DNA Methyltransferases and Ten-Eleven Translocation Enzymes are Detected in the Epididymis and Maturing Spermatozoa

**FIGURE 6:**
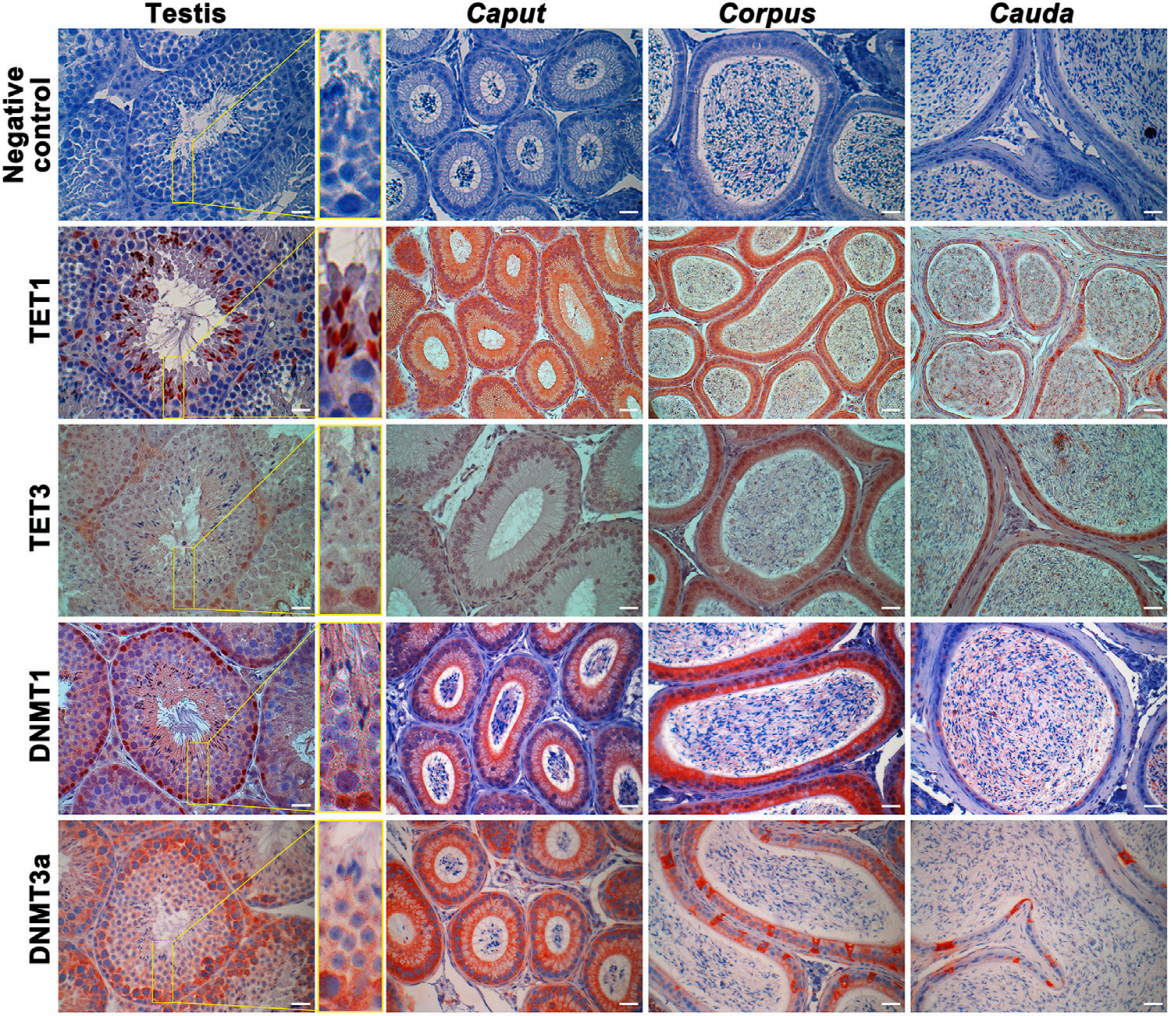
Immunohistochemical detection of TET1, TET3, DNMT1, and DNMT3a in the testis, *caput*, *corpus*, and *cauda* epididymis. Omission of primary antibodies were used as a negative control. Results are representative of three independent experiments. Insets (yellow square) represent ×3 magnification. Scale bar: 30 μm.

**FIGURE 7:**
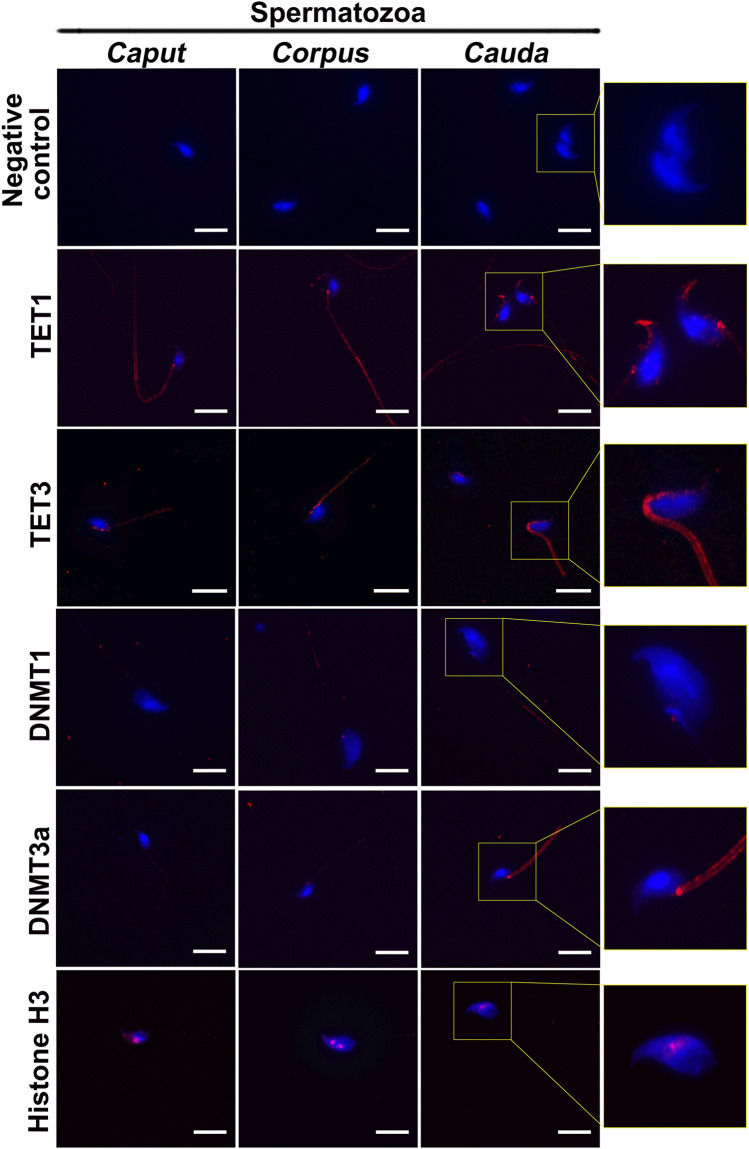
Immunofluorescence detection of TET1, TET3, DNMT1, and DNMT3a in c*aput, corpus and cauda* spermatozoa. Detection of Histone H3 was used as a positive control for the detection of sperm nucleic proteins (arrow). Omission of primary antibodies was used as a negative control. Insets (yellow squares) represent ×2 magnification of spermatic heads. Results are representative of three independent experiments. Scale bar: 10 μm.

DNA methyltransferases (DNMTs) and ten-eleven translocation (TET) enzymes control the dynamics of DNA epigenetic marks through CpG dinucleotide methylation and demethylation, respectively (Rasmussen and Helin, 2016; Edwards et al., 2017). In order to determine the origin of methylation changes observed in the maturing spermatozoa genome, we explored the expression of these enzymes in the epididymis. Both enzyme types, including DNMT1, DNMT3a, TET1, and TET3, were detected by immunofluorescence staining and immunohistochemistry in the post-testicular environment (Figures 6, 7). Consistent with their close interaction with DNA moieties during spermatogenesis, most of these enzymes were found in the nuclear compartments of germ cells at different developmental stages (Figure 6). For instance, while DNMT1 was detected in the nucleus of spermatogonia, TET1 was detected in the nucleus of round and elongated spermatids. Moreover, the expression of TET3 was observed in the nucleus of all testicular germ cells (Figure 6). In contrast, DNMT3a was the only enzyme detected in the cytoplasmic fraction of germ cells during spermatogenesis. While these enzymes could not be detected in epididymal spermatozoa by immunohistochemistry (Figure 6), further investigations were performed by immunofluorescence on spermatozoa from different epididymal segments (Figure 7). TET 1 was found in the peri-nuclear fraction and connecting piece of *caput*, *corpus,* and *cauda* spermatozoa as well as in the sperm flagellum, whereas TET3 detection was restricted to the sperm midpiece in all epididymal segments. Furthermore, DNMT3a displayed a strong signal in spermatozoa from the cauda epididymidis, and DNMT1 was weakly observed along the flagellum. Therefore, despite the successful detection of Histone H3 that confirmed the accessibility of nucleic antigens under our treatment conditions, none of the DNMT or TET enzymes were detected in the sperm nucleus compartment (Figure 7). However, TET1 and DNMT3a were successfully detected by western blot in positive control testis extracts, as well as in epididymal fluid (DNMT3a) and spermatozoa extracts (TET1) **(** Supplementary Figure S4), suggesting a potential role for these enzymes in the dynamics of sperm methylation profiling along the epididymis.

### Different DNMT and TET Enzyme Activity Profiles were Found in the Epididymal Fluid and Spermatozoa

**FIGURE 8:**
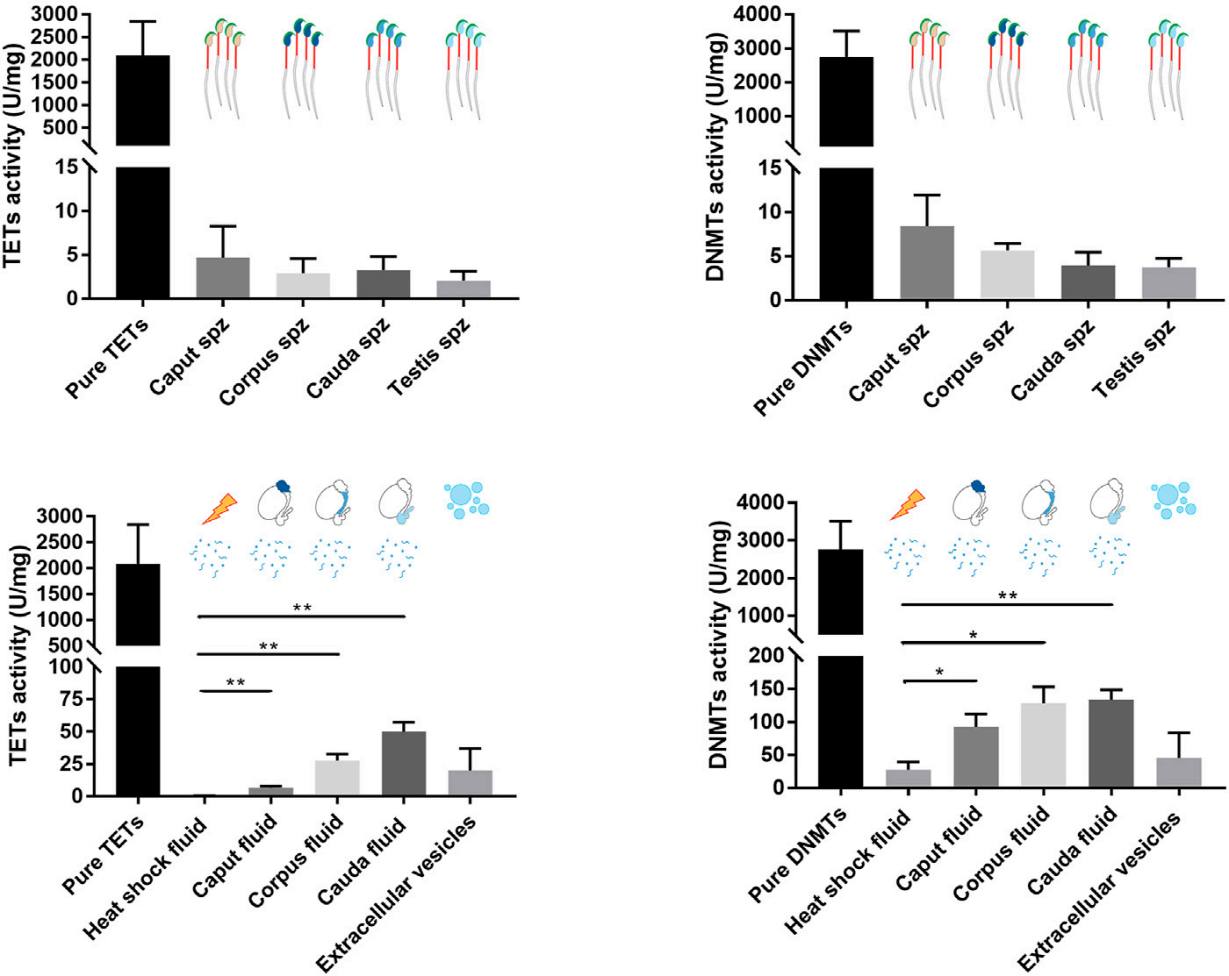
TET and DNMT enzyme assays performed on spermatozoa, epididymal fluid, and extracellular vesicle samples. Nuclear protein extracts from *caput*, *corpus*, and *cauda* spermatozoa, and epididymal fluid from different segments were used for TET and DNMT enzyme assays. Results are representative of three independent experiments. Total enzyme activities were detected in each sample (mean ± SEM). Pure enzymes and *cauda* epididymal fluid denatured by heat shock were used as positive and negative controls, respectively. Spz: spermatozoa. * and ** represent significant differences *p* < 0.05 and <0.005, respectively.

The activity levels of DNMT and TET enzymes were determined by enzyme assay to further determine their roles in spermatozoa and sperm-surrounding fluid in the post-testicular environment (Figure 8). Since epididymal fluid contains a fraction of extracellular vesicles (EVs) that allows the transfer of enzymes to maturing spermatozoa (Krapf et al., 2012), EV-free epididymal fluid was separated from EV-containing fractions in this study. While DNMT and TET activities were detected at slightly above threshold values in sperm nuclei extracts (4–8 ± 4 U/mg for DNMTs and 2 to 5 ± 3 U/mg for TETs), stronger DNMT activities ranging from 80 to 120 ± 30 U/mg and 10 to 30 ± 20 U/mg for TET activities were detected in epididymal fluid fractions devoid of EVs. DNMT and TET activities were significantly higher in fluid from the different epididymal segments compared with the heat-inactivated negative control, confirming the presence of methylation and demethylation activities in the sperm-surrounding fluid in these segments. No enzyme activity was detected in the EV fractions, which was comparable to observations for the enzyme-inactivated negative control (Figure 8), demonstrating that active DNMT and TET enzymes are not delivered to maturing spermatozoa by EVs.

### Sperm DNA Methylation Changes Observed in the Epididymis are not Controlled by DNMT and TET Enzymes

**FIGURE 9:**
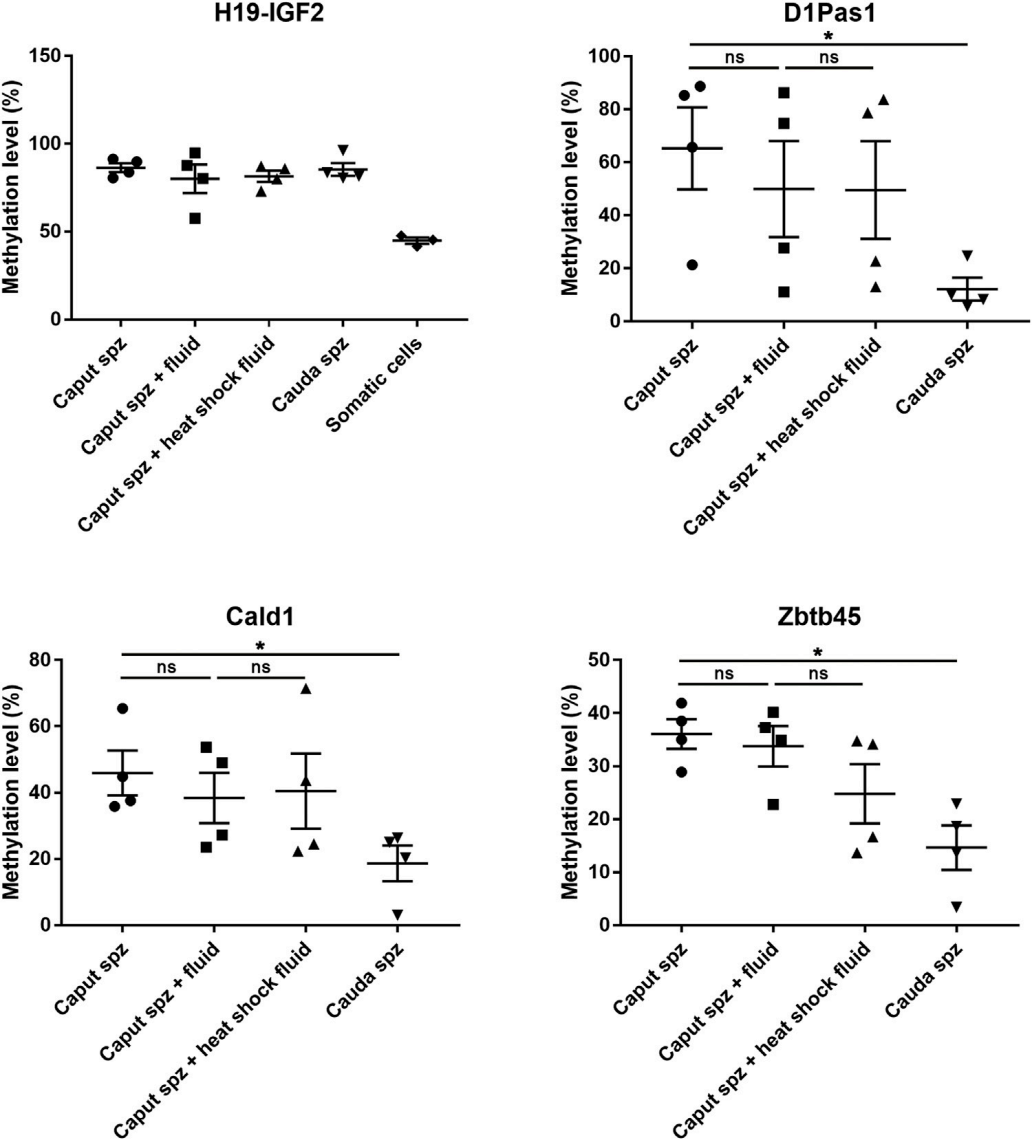
Effect of epididymal fluid on sperm methylation profiling. Following incubation of *caput* spermatozoa with *cauda* epididymal fluid or enzyme-inactivated fluid (heat shock), DNA loci that exhibited significant methylation changes during post-testicular maturation were analyzed by pyrosequencing. For each locus, four samples of testicular, *caput*, *corpus*, and *cauda* spermatozoa were used. H19-IGF2 served to validate the absence of somatic cell contamination. *p-value < 0.05.

In order to investigate whether the sperm methlylation profile can be modified following contact with sperm microenvironmental conditions, i.e., the epididymal fluid, we conducted *caput* spermatozoa/*cauda* epididymal fluid co-incubation assays for 2 hours at 37°C (Figure 9). Various DNA loci displayed significant methylation changes during post-testicular sperm maturation (Figure 4); however, no significant methylation variation was detected for Cald1 or Zbtb45 in the presence of *cauda* epididymal fluid (Figure 9). Although a slight modification of D1Pas1 methylation level was detected by pyrosequencing following incubation with *cauda* epididymal fluid (15%), this was also present when proteins were denatured prior to co-incubation with spermatozoa (Figure 9). Overall, this shows that enzymes from the sperm-surrounding fluid do not account for sperm DNA methylation changes observed at the post-testicular level.

### Sperm Heterogeneity Accounts for Sperm DNA Methylation Changes Observed in the *Caput* Epididymidis

**FIGURE 10:**
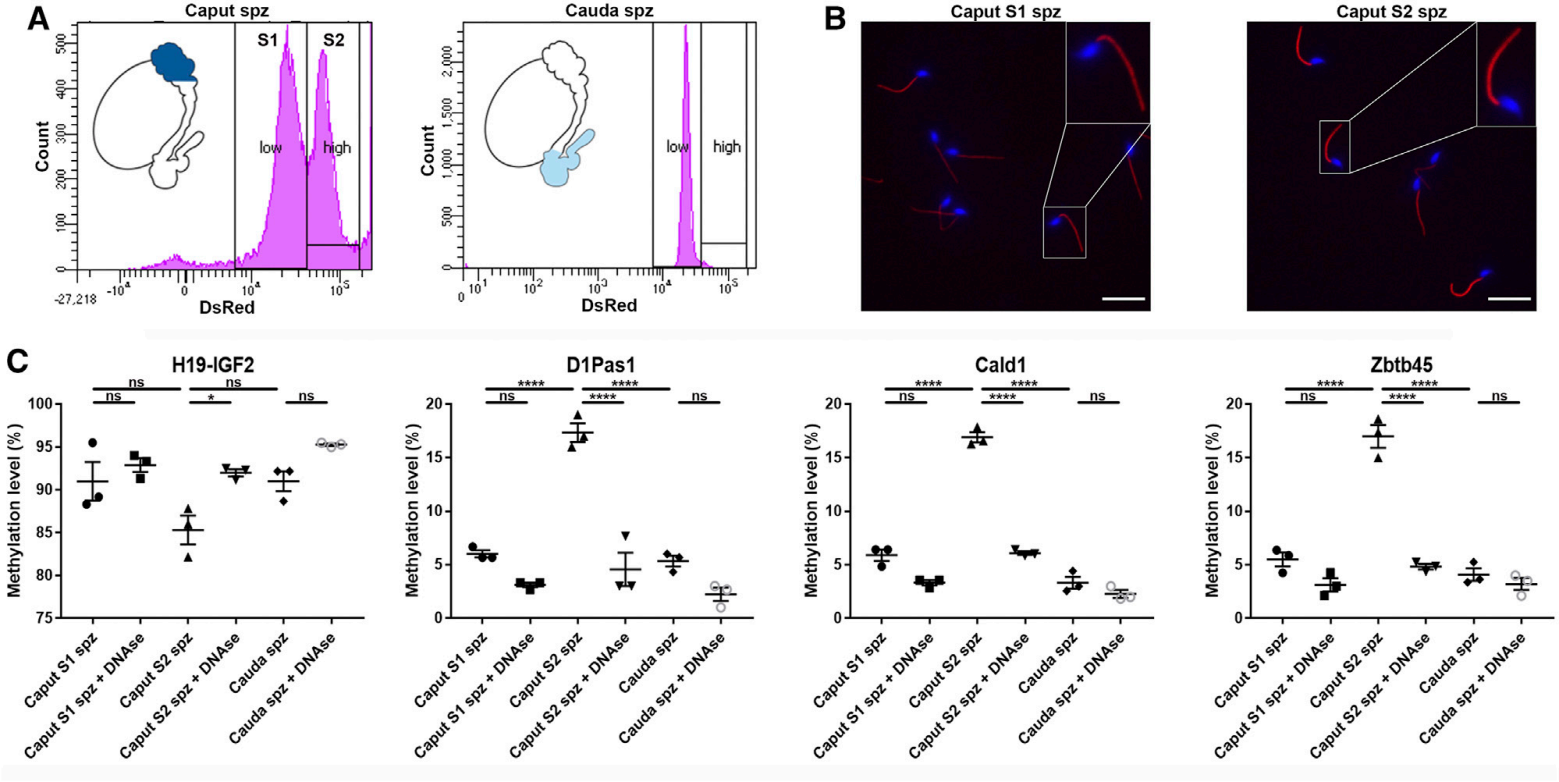
Methylation profiles of D1Pas1, Cald1 and Zbtb45 in caput epididymal sperm with low and high DsRed intensity levels. **(A)** Sperm subpopulations were analyzed by FACS according to their endogenous DsRed intensity levels (S1: subpopulation with low DsRed intensity, S2: subpopulation with high DsRed intensity) and **(B)** observed under a fluorescent microscope. Insets (yellow squares) represent ×2 magnifications of spermatic heads. Scale bar: 20 μm **(C)** Prior to sperm DNA extraction and pyrosequencing on H19-IGF2, D1Pas1, Cald1 and Zbtb45 loci, sperm subpopulations were treated or not with DNase I (+DNAse). H19-IGF2 served to assess the presence of somatic cell contamination. Spz: spermatozoa; *, **, and **** indicate *p*-values of *p* < 0.05, <0.005, and <0.0001 respectively.

According to their DsRed endogenous intensity, two subpopulations of spermatozoa were detected in the *caput* epididymidis and, to a lower extent in the *corpus* epididymidis: low intensity DsRed (S1) vs. high intensity DsRed (S2) (Figures 2A, 10A), the proportion of S1 and S2 populations being variable between mice **(** Supplementary Figure S6). In comparison, spermatozoa isolated from the *cauda* epididymidis display a strong homogeneity in terms of DsRed intensity level (Figure 10A). Considering that intra-individual changes in spermatozoa DNA methylation profiles have been reported between sperm sub-populations (Barzideh et al., 2013; Capra et al., 2019; Jenkins et al., 2015), we examined whether spermatozoa with low *vs.* high DsRed endogenous intensity harbored different DNA methylation profiles that could account for epigenetic changes observed in the *caput* epididymidis (Figure 10). While S1 and S2 populations isolated by FACS indeed displayed differences in terms of DsRed fluorescence intensity, they shared a similar morphology (Figure 10B). Furthermore, the exclusive detection of spermatozoa by fluorescent microscopy (Figure 10B) and the hypermethylation level of sperm DNA on H19-IGF2 locus (>80%) (Figure 10C) confirmed the absence (or the negligible presence) of somatic cells from FACS-sorted sperm populations. While significant methylation changes were detected on D1Pas1 (11.4 ± 1.2%), Cald1 (11.0 ± 0.6%), and Zbtb45 (11.5 ± 0.9%) loci between S1 and S2 sperm populations, no significant changes were observed between S1 *caput* sperm population and *cauda* spermatozoa, suggesting that the presence of S2 sperm population is accountable for the major methylation changes observed at the post-testicular level (Figure 10C). Considering that the presence of extracellular DNA was shown to affect sperm methylation profiling analysis (Galan et al., 2021), *caput* S1, S2 and *cauda* sperm populations were treated with DNase I prior to DNA extraction and pyrosequencing. Methylation levels detected on D1Pas1, Cald1, and Zbtb45 loci from S2 sperm population, was significantly reduced to a level equivalent to S1 sperm population after DNAse treatment, suggesting that caput S2 subpopulation was associated with epididymal extracellular DNA. At the contrary, the methylation levels of S1 and cauda sperm populations remained unchanged after treatment (Figure 10C). While we hypothesized that sperm populations associated with extracellular DNA might correspond to defective/apoptotic spermatozoa, annexin-V assay showed no correlation between sperm apoptosis and DsRed intensity level (data not shown). Furthermore, no significant changes were detected between annexin-V negative and annexin-V positive *caput* spermatozoa (<10%) (Supplementary Figure S5), indicating that the different methylation patterns observed between S1 and S2 were not associated with annexin-V positive defective/apoptotic spermatozoa.

## Discussion

In addition to their role of transporting the paternal genome to the site of fertilization, spermatozoa transfer epigenetic information to the oocyte that influences embryo development and progeny outcome (Sharma, 2019; Ben Maamar et al., 2021; Yin et al., 2021). While sperm DNA methylation occurrence has primarily been described during spermatogenesis, a limited number of studies have addressed the cytosine methylation changes that may affect spermatozoa during their maturation in the epididymis (Ariel et al., 1994; Ben Maamar et al., 2019; Galan et al., 2021). To overcome potential somatic cell and extracellular DNA contamination that may have previously impeded the specificity of sperm methylome profiling, we determined the sperm methylome from FACS-isolated fluorescent spermatozoa purified at different stages of development/maturation. Combined with a genome-wide RRBS approach, we observed a transitory change in sperm DNA methylation profile in the proximal region of the epididymis and investigated the mechanisms by which such epigenetic modifications could occur.

Inspired by the study from Ariel et al. (Ariel et al., 1994) that substantiated the remethylation of three spermatogenesis-specific genes, i.e., Pgk–2, ApoA1 and Oct–3/4 at the post-testicular level, we determined the genome-wide methylation profile of four sperm populations isolated from the testis, *caput*, *corpus*, and *cauda* epididymidis. Although our sequencing results (RRBS and pyrosequencing) did not show any methylation changes to these specific genes, we detected a transitory wave of hyper-/hypomethylation in the proximal segment of the epididymidis, which is consistent with the recent whole-genome bisulfite sequencing (WGBS) study from Galan et al. (Galan et al., 2021). Comparably to the latter study we found a similar methylation variation rate, with 1835 hypomethylated DMRs (45%) and 2238 hypermethylated DMRs (55%) in the sperm population isolated from the *caput* epididymis compared with testicular spermatozoa (100 bp, methylation difference >25%). In addition, 55% of hypomethylated genes (824 genes) implicated in the regulation of transcription from RNA polymerase II promoter, multicellular organism development, regulation of cell proliferation and migration, and 52% of hypermethylated genes (924 genes) associated with multicellular organism development, regulation of transcription, cell adhesion and differentiation were found susceptible to methylation changes in both studies. However, none of these common changes were detected on the same loci. According to previous studies, the use of diverse mouse strains [FVB vs. B6D2F1- Tg (CAG/su9-DsRed2, Acr3-EGFP)] (Grimm et al., 2019), and methylation analysis approaches (WGBS vs. RRBS and pyrosequencing) (Bonora et al., 2019) could account for these differences. In addition, the different methods used to isolate sperm populations (discontinuous Percoll gradient on squeezed tissues vs. FACS and intraluminal perfusion) may influence the purity of the samples and their potential association with extracellular DNA and/or somatic cells (Galan et al., 2021). Herein the isolation of spermatozoa from the CAG/su9-DsRed2, Acr3-EGFP transgenic mouse model was optimized through the selection of spermatids and spermatozoa presenting an intact acrosome.

Given that sperm DNA is highly compacted due to the replacement of histones by protamines and the formation of disulfide bridges between their thiol groups (Conrad et al., 2005; Hutchison et al., 2017), cytosine accessibility and the mechanisms by which its methylation occur remain to be established. While the majority of histones are replaced by the end of spermatogenesis, protamine thiol oxidation and molecular cross-links occur sequentially as spermatozoa mature in the epididymis, with the highest proportion of decondensed DNA being found in the proximal epididymidis (Conrad et al., 2005). Therefore, the window of hypo- and hypermethylation we observe in the caput epididymidis coincides with highest cytosine accessibility in this segment compared with the more distal regions. This suggests the presence of a higher susceptibility of spermatozoa to methylation changes in the proximal *vs.* distal epididymidis, which is consistent with the study from Ben Maamar et al., 2019 who showed the greatest proportion of DMR localized to *caput* spermatozoa following DDT exposure (Ben Maamar et al., 2019). However, the absence of correlation between sperm DNA methylation changes and the activity of DNMT and TET enzymes detected in epididymal spermatozoa and their surrounding fluid suggests that (de)methylase activity does not account for these transitory modifications. This is further supported by the absence of DNMTs and TET enzymes in sperm nucleus. In accordance with (Marques et al., 2011; Ni et al., 2016), while we detected TET1, TET3 and DNMT3a enzymes in sperm extra-nuclear compartments, it is possible that an active mechanism is in place in spermatozoa to retain these enzymes outside the nucleus to prevent binding and modification of DNA substrate, as observed in early embryo (Cardoso and Leonhardt, 1999).

The epididymis has been proposed to be a site where heterogeneous populations of spermatozoa are subjected to a quality control through the removal of defective/apoptotic spermatozoa (Sutovsky et al., 2001; Sutovsky, 2003; Ramos-Ibeas et al., 2013), possibly under the surveillance of monocyte macrophages that populate the proximal epididymidis (Battistone et al., 2020; D'Amours et al., 2012a; D'Amours et al., 2012b; Da Silva et al., 2011; Wang et al., 2021). Here, we detected two sperm populations from CAG/su9-DsRed2, Acr3-EGFP transgenic mice displaying distinct methylation profiles in the *caput* epididymidis, where a mechanism for recognizing defective spermatozoa takes place (Axner, 2006; D'Amours et al., 2012b; Ramos-Ibeas et al., 2013; Sutovsky, 2003; Sutovsky et al., 2001). These two populations can be discriminated based on their level of DsRed2 endogenous fluorescence, which is ubiquitously expressed under the CAG promoter and detected in mitochondria under the control of an import signal sequence (Atp5g1). Considering that the ATP synthase component Atp5g1 participates in the control of oxidative phosphorylation and that sperm methylation is susceptible to the presence of reactive oxygen species (ROS) (Agarwal et al., 2006; Tunc and Tremellen, 2009), it is possible that a subpopulation of spermatozoa possessing a higher ATP synthase activity would increase the production of ROS and, therefore, methylation changes. Furthermore, our results show that the sperm population displaying the highest intensity of DsRed corresponds to a sperm population whose methylation profile is susceptible to DNAse treatment, suggesting its association with extracellular DNA. Whether this subpopulation corresponds to apoptotic/defective spermatozoa entrapped in immune cells derived DNA (e.g., Neutrophil extracellular traps (NETs) or Macrophage Extracellular Traps (METs) remains to determine. In line with the study from (Galan et al.) it is possible that extracellular DNA binds to defective spermatozoa as a process of epididymal sperm quality control and accounts for some differences observed at the epigenetic level in the proximal epididymidis.

In conclusion, the isolation of endogenously labeled spermatozoa enabled us to confirm that a transitory window of sperm-associated epigenetic mark changes is predominantly observed in the proximal epididymis, in part due to the presence of extracellular DNA in this segment. A better understanding of the origin of sperm heterogeneity in the epididymis and its degree of conservation in human will eventually provide new considerations regarding sperm selection procedures used in fertility clinics.

## Data Availability

The datasets presented in this study can be found in online repositories. The names of the repository/repositories and accession number(s) can be found below: https://www.ncbi.nlm.nih.gov/geo/, accession # GSE191051.
