# Supplementary material for: Sperm Heterogeneity Accounts for Sperm DNA Methylation Variations Observed in the Caput Epididymis, Independently From DNMT/TET Activities
Source: Front Cell Dev Biol. 2022 Mar 22;10:834519. doi: 10.3389/fcell.2022.834519 (PMC8981467; doi:10.3389/fcell.2022.834519)
Supplement: Supplementary file 1 [file DataSheet2.docx]

**Supplemental materials**


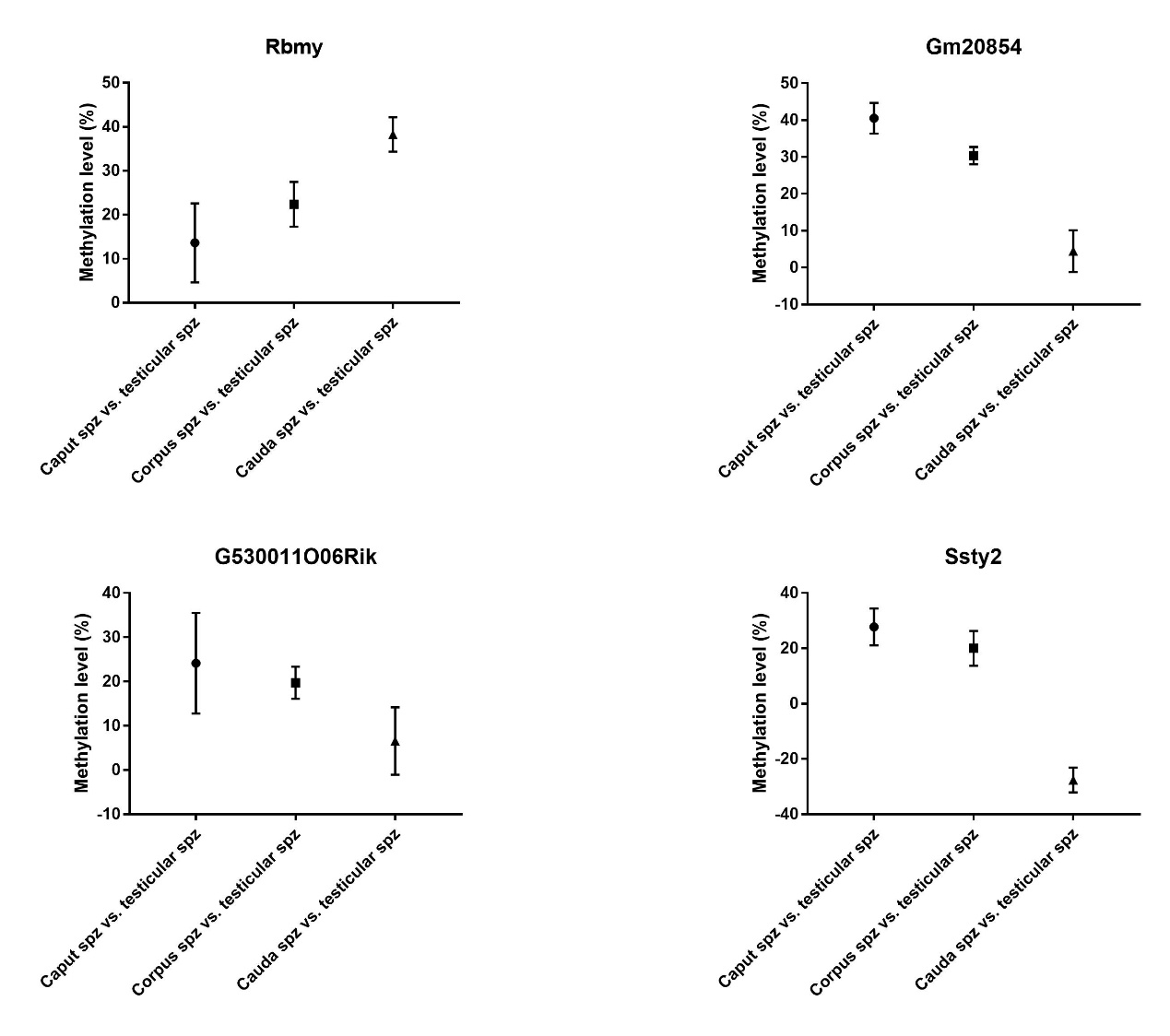


**Supplemental figure 1. Methylation changes of Y chromosomes in spermatozoa during the post-testicular maturation.** Four genes that presented methylation changes have been identified by RRBS. Spz: spermatozoa


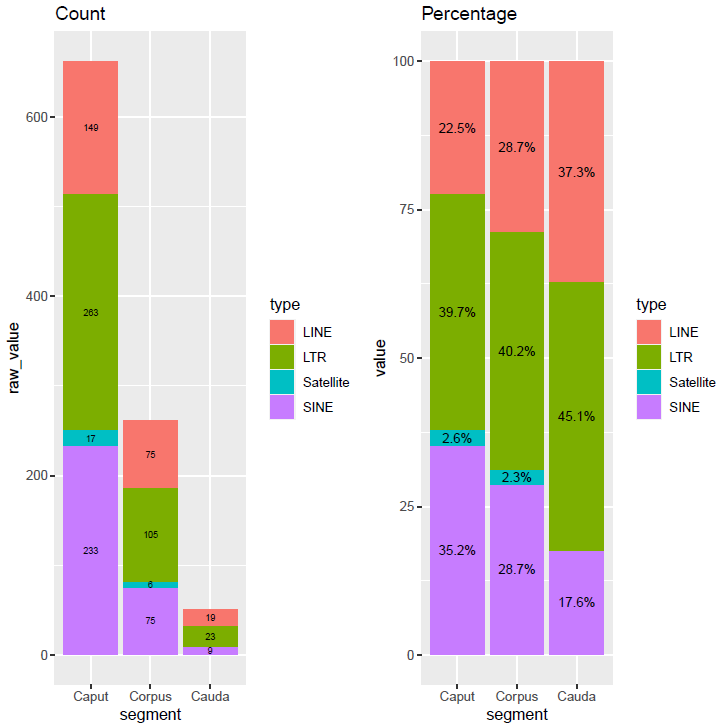


**Supplemental Figure 2. Sperm DNA methylation alteration on repetitive regions.** Histogram bar illustrating the proportions of CpGs covered by RRBS in repetitive regions annotated as short interspersed nuclear element (SINE) (purple), satellite (blue), long terminal repeat (LTR) (red), and long interspersed nuclear element (LINE) (green).


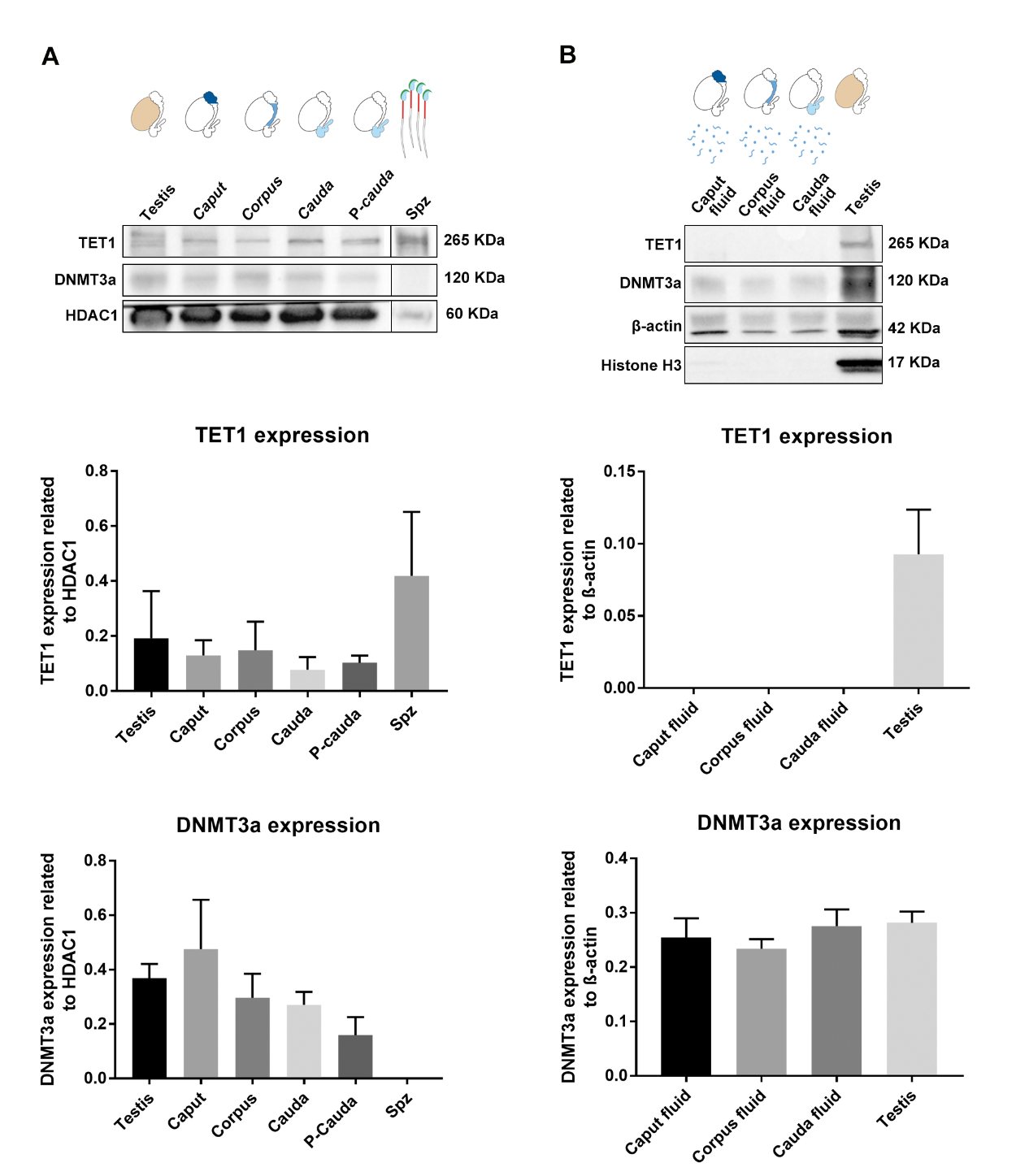


**Supplemental Figure 3.** **Quantification of TET1 and DNMT3a expression in the testis, *caput*, *corpus*, *cauda* epididymis, spermatozoa, and epididymal fluid by Western-blot.** Results are representative of three independent experiments. HDAC1 and β-Actin was used as an internal loading control. In addition, histone H3 was used to validate non-cellular contamination in the epididymal fluid.Cauda epididymis was perfused (P-Cauda) to remove spermatozoa and epididymal fluid from the sample. TET1 and DNMT3a expression levels were normalized to HDAC1 and β-Actin (mean ± SEM). Spz: spermatozoa sample.


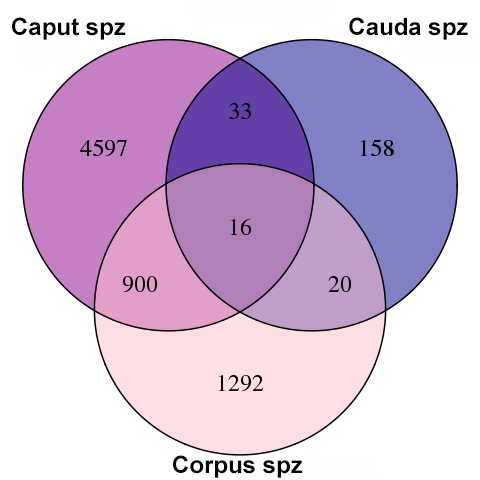


**Supplemental figure 4. Overlap analysis of differentially methylated sites among caput, corpus, and cauda spermatozoa.** Spz: spermatozoa


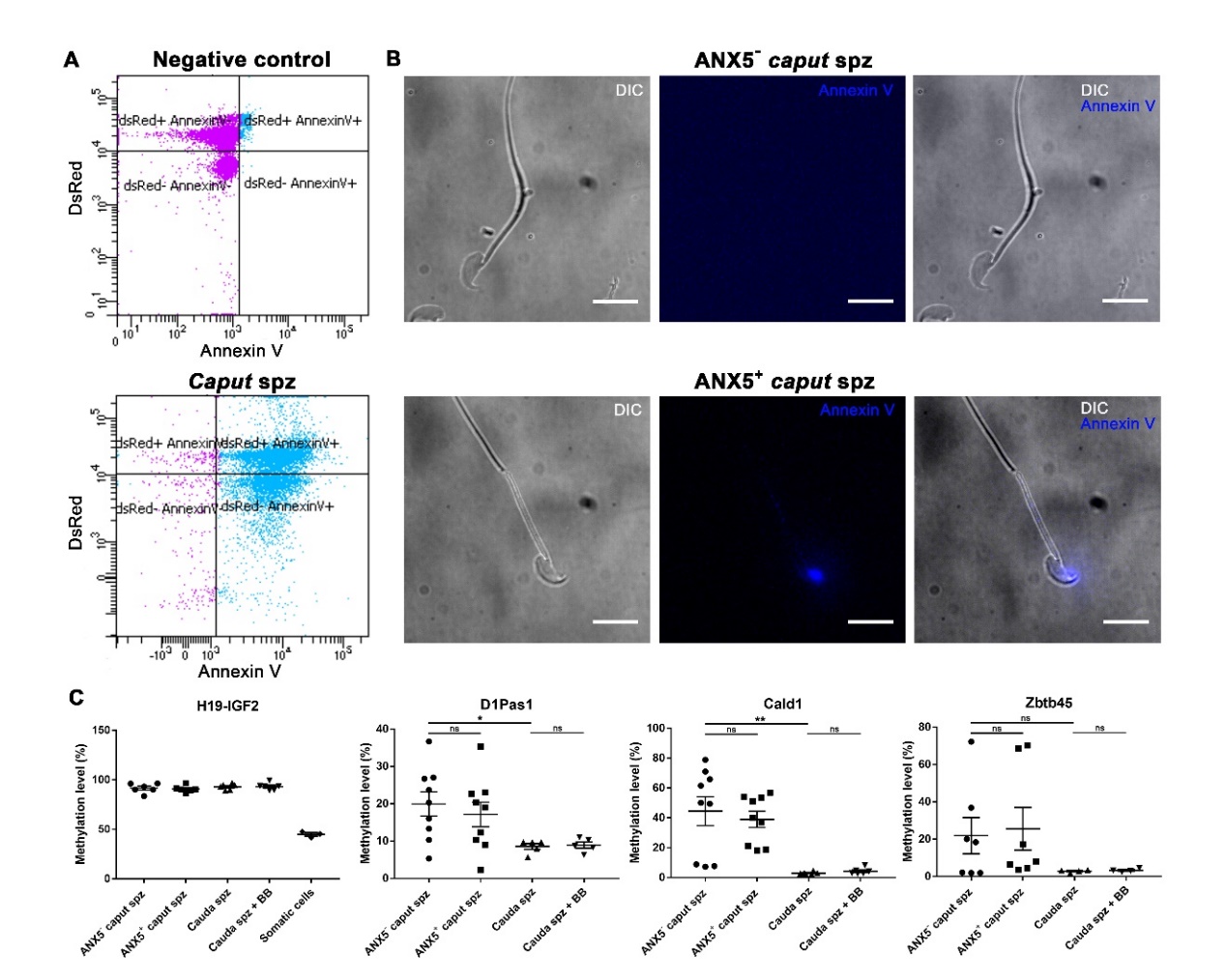


**Supplemental figure 5. Methylation profiles of D1Pas1, Cald1 and Zbtb45 in annexin-V positive and annexin-V negative spermatozoa.** **(A)** Annexin-V positive and negative spermatozoa were isolated by FACS. Spermatozoa collected from the cauda epididymidis, where few defective/apoptotic spermatozoa are found, served as a negative control for annexin-V detection. **(B)** The purity of annexin-V positive and negative spermatozoa was assessed by fluorescence microscopy. **(C)** DNA loci that exhibited significant methylation changes during post-testicular maturation were analyzed by pyrosequencing. For each locus, four samples of *caput* annexin-V positive and annexin-V negative spermatozoa, *cauda* spermatozoa, and *cauda* spermatozoa incubated with annexin-V binding buffer (BB)(control condition) were used. H19-IGF2 served to validate the absence of somatic cell contamination. Spz: spermatozoa; ANX5-: annexin-V negative; ANX5+: Annexin-V positive; BB: annexin-V binding buffer. Scale bar: 10 μm. * and ** indicate respective p-values of p < 0.05 and p < 0.005


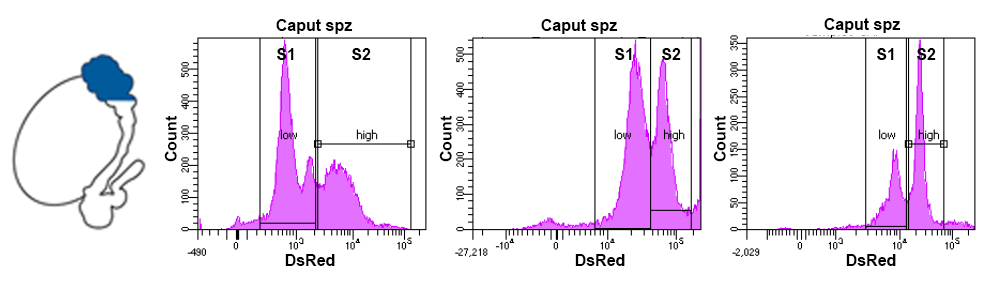


**Supplemental figure 6. FACS analysis of caput sperm subpopulations from different mice.** Three typical distributions of caput sperm subpopulations were analyzed depending on DsRed intensity level. S: subpopulation; spz: spermatozoa
