# Supplementary material for: Sperm Heterogeneity Accounts for Sperm DNA Methylation Variations Observed in the Caput Epididymis, Independently From DNMT/TET Activities
Source: Front Cell Dev Biol. 2022 Mar 22;10:834519. doi: 10.3389/fcell.2022.834519 (PMC8981467; doi:10.3389/fcell.2022.834519)
Supplement: Supplementary file 2 [file DataSheet1.docx]

**Supplementary table 1: List of pyrosequencing primers used for sperm DNA methylation changes analysis.**

| Region of interest | Sequence |
| --- | --- |
| H19 F1 | AGGGGGGTAGGATATATGTAT |
| H19 R1 Bio | AACTAACATAAACCCCTAACCTCA |
| H19 Seq | GGATTAGGATTTTTAAATTAATAAG |
| ApoA1 F1 | GGGGAGGTAAGTGTTGTTATT |
| ApoA1 R1 Bio | CACACAATTCAAACCCATAAAAACATTAA |
| ApoA1 Seq | GGTAAGTGTTGTTATTTGT |
| D1Pas1 F1 | AGGTAGAAGTGGTTTTGGTAAATT |
| D1Pas1 R1 Bio | TCCCATAATAATCTCTCCCATCTC |
| D1Pas1 Seq | AGTGGTTTTGGTAAATTT |
| Capn2 F1 | ATGGAGGAAAGAATTTGTTAATTATAAGT |
| Capn2 R1 Bio | CATCCCCCCTCTTAACACACAATTCA |
| Capn2 Seq | GTAGAGTATATTATTATTTTGTTGG |
| Ldb3 F1 | GGAGGATTAGGGAGTTGAGAA |
| Ldb3 R1 Bio | ACTTAACCCCTCAACTCCTA |
| Ldb3 Seq | GTTAAATGAGGAGAGAGTATTTTT |
| Cald1 F1 | GGAAGTTGAAGGTGTTTTATAAGT |
| Cald1 R1 Bio | CTTCTCTCTTCCCTCTTTTTCT |
| Cald1 Seq | GGAAGTAGGTAAGAGGTT |
| Zbtb45 F1 | TGGGGGTGTAGGTAGTTGA |
| Zbtb45 R1 Bio | CACCCCTACCCCCTCCACT |
| Zbtb45 Seq | GGGTGTAGGTAGTTGAA |

**Supplementary table 2: List of antibodies used for Western blot, Immunohistochemistry, and Immunofluorescence.**

| Primary antibody | | | |
| --- | --- | --- | --- |
| Application | Dilution | name | reference |
| WB, IHC, IF | 1:2000, 1:200, 1:200 | Tet1 | GTX12407 (GeneTex) |
| IHC,IF | 1:200, 1:200 | Tet3 | GTX121453 (GeneTex) |
| IHC,IF | 1:50, 1:50 | Dnmt1 | Ab19905 (Abcam) |
| WB, IHC, IF | 1:500, 1:100, 1:100 | Dnmt3a | Ab13888 (Abcam) |
| WB | 1:2000 | HDAC1 | Ab19845 (Abcam) |
| WB | 1:5000 | β-actin | A5441 (SIGMA) |
| WB,IF | 1:2000, 1:200 | Histone H3 | Ab1791 (Abcam) |
| IF | 1:200 | Anti-Histone H3 (citrulline R2 + R8 + R17) | Ab5103 (Abcam) |
| WB | 1:2000 | HBB (Hemoglobin) | Ab231732 (Abcam) |
| Secondary antibody | | | |
| WB | 1:5000 | GAM(HRP) | Ab97023 (Abcam) |
| WB | 1:5000 | GAR(HRP) | Ab97051 (Abcam) |
| IHC | 1:200 | GAM(Biotin) | 115-061-166 (IR) |
| IHC | 1:200 | GAR(Biotin) | 111-065-144 (IR) |
| IF | 1:200 | DAM647 | 715-605151 (IR) |
| IF | 1:200 | DAR647 | 711-605-152 (IR) |
